# Tracking smell loss to identify healthcare workers with SARS-CoV-2 infection

**DOI:** 10.1371/journal.pone.0248025

**Published:** 2021-03-03

**Authors:** Julian J. Weiss, Tuki N. Attuquayefio, Elizabeth B. White, Fangyong Li, Rachel S. Herz, Theresa L. White, Melissa Campbell, Bertie Geng, Rupak Datta, Anne L. Wyllie, Nathan D. Grubaugh, Arnau Casanovas-Massana, M. Catherine Muenker, Adam J. Moore, Ryan Handoko, Akiko Iwasaki, Richard A. Martinello, Albert I. Ko, Dana M. Small, Shelli F. Farhadian, the Yale IMPACT Research Team

**Affiliations:** 1 Department of Medicine, Section of Infectious Diseases, Yale School of Medicine, New Haven, Connecticut, United States of America; 2 Department of Neurology, Yale School of Medicine, New Haven, Connecticut, United States of America; 3 Department of Psychiatry, Yale School of Medicine, New Haven, Connecticut, United States of America; 4 Department of Epidemiology of Microbial Diseases, Yale School of Public Health, New Haven, Connecticut, United States of America; 5 Yale Center for Analytical Sciences, Yale School of Public Health, New Haven, Connecticut, United States of America; 6 Department of Psychiatry and Human Behavior, Alpert Medical School of Brown University, Providence, Rhode Island, United States of America; 7 Department of Psychology, Le Moyne College, Syracuse, New York, United States of America; 8 SUNY Upstate Medical University, Syracuse, New York, United States of America; 9 Center for Outcomes Research and Evaluation, Yale-New Haven Health, New Haven, Connecticut, United States of America; 10 Yale School of Medicine, New Haven, Connecticut, United States of America; 11 Department of Immunobiology, Yale School of Medicine, New Haven, Connecticut, United States of America; 12 Howard Hughes Medical Institute, Chevy Chase, Maryland, United States of America; 13 Department of Pediatrics, Yale School of Medicine, New Haven, Connecticut, United States of America; 14 Department of Infection Prevention, Yale-New Haven Health, New Haven, Connecticut, United States of America; 15 Department of Psychology, Yale University, New Haven, Connecticut, United States of America; AUSL della Romagna, ITALY

## Abstract

**Introduction:**

Healthcare workers (HCW) treating COVID-19 patients are at high risk for infection and may also spread infection through their contact with vulnerable patients. Smell loss has been associated with SARS-CoV-2 infection, but it is unknown whether monitoring for smell loss can be used to identify asymptomatic infection among high risk individuals. In this study we sought to determine if tracking smell sensitivity and loss using an at-home assessment could identify SARS-CoV-2 infection in HCW.

**Methods and findings:**

We performed a prospective cohort study tracking 473 HCW across three months to determine if smell loss could predict SARS-CoV-2 infection in this high-risk group. HCW subjects completed a longitudinal, behavioral at-home assessment of olfaction with household items, as well as detailed symptom surveys that included a parosmia screening questionnaire, and real-time quantitative polymerase chain reaction testing to identify SARS-CoV-2 infection. Our main measures were the prevalence of smell loss in SARS-CoV-2-positive HCW versus SARS-CoV-2-negative HCW, and timing of smell loss relative to SARS-CoV-2 test positivity. SARS-CoV-2 was identified in 17 (3.6%) of 473 HCW. HCW with SARS-CoV-2 infection were more likely to report smell loss than SARS-CoV-2-negative HCW on both the at-home assessment and the screening questionnaire (9/17, 53% vs 105/456, 23%, *P* < .01). 6/9 (67%) of SARS-CoV-2-positive HCW reporting smell loss reported smell loss prior to having a positive SARS-CoV-2 test, and smell loss was reported a median of two days before testing positive. Neurological symptoms were reported more frequently among SARS-CoV-2-positive HCW who reported smell loss compared to those without smell loss (9/9, 100% vs 3/8, 38%, *P* < .01).

**Conclusions:**

In this prospective study of HCW, self-reported changes in smell using two different measures were predictive of SARS-CoV-2 infection. Smell loss frequently preceded a positive test and was associated with neurological symptoms.

## Introduction

It is increasingly clear that asymptomatic and pre-symptomatic infection play an important role in the ongoing spread of COVID-19 [1], with peak infectiousness likely occurring on or before symptom onset [2, 3]. Healthcare workers (HCWs) may be at an increased risk of SARS-CoV-2 infection due extended exposure to highly infectious patients. Up to 40% of HCWs are asymptomatic, representing an important chain of transmission to be further investigated [4]. There is therefore an urgent need for non-invasive screening tools to aid in early identification of infected individuals, including at-risk healthcare workers [5–7].

Olfactory impairment is a common manifestation of COVID-19, with meta-analyses indicating a prevalence of 77–86% in studies using standardized olfactory testing [8–11]. Among patients with COVID-19 assessed for smell loss, 41% and 98% had impaired tests of olfaction on two different tests of olfactory function [12, 13], suggesting that smell loss may aid in COVID-19 case identification [14]. While one recent study confirmed higher rates of self-reported smell loss in HCWs [15], smell loss has not yet been prospectively evaluated in uninfected individuals undergoing regular, frequent SARS-CoV-2 testing. Self-monitoring for the first sign of diminished smell function using objective measures would enable rapid testing and/or self-quarantine, thus preventing further spread prior to a formal diagnosis.

The aim of this study was to determine if tracking smell sensitivity and loss using an at-home assessment could identify HCW who are infected with SARS-CoV-2. Since testing for objective smell loss using standard laboratory or clinical techniques is not feasible for widespread testing, we employed a brief at-home smell sensitivity screen, the Yale Jiffy (**S1 Table**), to track ratings of odor intensity perception using two household olfactory stimuli. High risk HCW undergoing routine (every 3 days) viral screening for SARS-CoV-2 infection were assessed for self-reported smell loss, along with symptoms commonly used to screen for COVID-19.

## Methods and materials

### Study setting, population, and recruitment

We conducted a prospective cohort study of smell symptomology nested within the Implementing Medical and Public Health Action against Coronavirus (CT) (IMPACT) study at Yale University (HIC # 200027690, approved by the Yale University Institutional Review Board). The goal of the parent study was to prospectively follow SARS-CoV-2-negative HCW at high risk of acquiring infection due to occupational exposures. The study recruited HCW working in the medical ICU or dedicated COVID-19 units at Yale New Haven Hospital (YNHH), a 1,541-bed tertiary care hospital located in New Haven, CT, USA. All participants provided written and/or verbal informed consent. Inclusion criteria for the IMPACT study included: a) aged 18 or older; b) English-speaking; c) working in a health care facility (YNHH, Yale Health); d) possible moderate to high risk exposure to COVID-19, or work in a COVID-19 unit, and e) SARS-CoV-2 negative at study entry. For this analysis of smell alterations and COVID-19, we excluded subjects without at least one SARS-CoV-2 PCR result and those who had not completed at least one daily symptom questionnaire or Yale Jiffy. All reported data were collected between March 31 and July 7, 2020.

### Viral testing

SARS-CoV-2 real-time quantitative polymerase chain reaction (RT-qPCR) testing was performed on self-collected nasopharyngeal and saliva specimens every three days. Specimens were processed and tested on the same day as collection following previously described protocols [16, 17]. Two HCW reported positive results from a CLIA-certified lab outside of the study protocol.

### Yale Jiffy

The Yale Jiffy is an online survey developed to screen for smell loss that can be conducted in under two minutes using readily available household items. The questionnaire includes two sections using self-ratings of: 1) ability to smell, and 2) strength of smell in response to olfactory and trigeminal stimuli. Peanut butter (or jam/jelly) was used as the olfactory stimulus as it has minimal or no trigeminal component, allowing for isolation of effects on the olfactory system. We also included a stimulus with a trigeminal component as a control stimulus (i.e., vinegar).

First, participants were asked to rate their ability to smell on a categorical scale (Poor/Average/Good/Very Good), to report any reduction in smell in the past week on a categorical scale (None/Slight/Moderate/Severe), and to rate the degree of reduction on a 10cm (0.0–10.0) visual analog scale (VAS). Next, participants were asked to hold the olfactory stimulus one inch from their nose and to provide ratings of both the strength of smell (0.0–10.0) and how different it smells from normal (0.0–10.0). HCW then held the trigeminal stimulus one inch from their nose and rated strength of sensation of irritation and difference from normal, as above. Since olfactory sensitivity fluctuates across the day [18], HCW were asked to complete the survey at approximately the same time each day using the same stimuli each time they completed the test. Responses were collected using Qualtrics, a secure HIPAA-compliant web-based survey platform, and retention was encouraged using daily e-mail reminders.

### Daily symptom questionnaire

As part of the IMPACT study, HCW completed an online daily symptom questionnaire. HCW were given a list of symptoms and prompted to indicate whether they had developed such symptoms in the past 24 hours. Listed symptoms included objective fever (>100.4°F), subjective fever, cough, shortness of breath, stuffy nose, sore throat, chills, sweating, malaise, fatigue, muscle pain, anorexia, nausea, vomiting, diarrhea, abdominal pain, and dizziness. In addition, we included screening questions for parosmia–changes in odor quality perception [19]–and for hypogeusia–reduced ability to taste [20]. Participants completed four parosmia screening questions (**S2 Table**), indicating how often they were bothered by common complaints caused by smell distortions, with responses ranging from “always” (1 point) to “never” (4 points). Parosmia was defined as a cumulative score less than or equal to 14 (out of 16). HCW responded to four hypogeusia screening questions for saltiness, sourness, sweetness, and bitterness, by indicating that they could detect these tastes “Easily” (3 points), “Somewhat” (2 points), or “Not at all” (1 point). Hypogeusia was defined as any total score less than the maximum of 12 points.

### Statistical analyses

Descriptive statistics were used to characterize the study population. The Fisher’s exact and Wilcoxon rank-sum tests were used to compare SARS-CoV-2-positive and negative HCW. Multivariable logistic regression models were developed to calculate adjusted odds ratios for the associations between smell symptoms and SARS-CoV-2 infection. Each model included one symptom as the predictor and additionally adjusted for age, sex, body mass index (BMI), ethnicity, and number of symptom surveys completed.

To further examine the strength of association between symptoms of smell loss and SARS-CoV-2 infection, we compared HCW ever reporting smell loss to a subset of HCW never reporting smell loss who were well-matched on age, sex, ethnicity, and the number of daily symptom surveys completed. We used the R package ‘MatchIt’ for this analysis [21], specifying a control to case ratio of 2:1 and employing optimal matching, which minimizes the overall differences between cases and controls. To estimate the strength of association between smell loss and SARS-CoV-2 infection, we conducted conditional logistic regression using the matched dataset and the R package ‘survival.’ We compared results of three matched logistic regression analyses among those ever reporting smell loss on either survey (Yale Jiffy or daily symptom questionnaire), on the Yale Jiffy only, and on the daily symptom questionnaire only. Sensitivity analyses tested whether inclusion of covariates was needed to adjust for residual confounding after matching, determined by a change in the point estimate of >10%.

Finally, we summarized responses to the Yale Jiffy and daily symptom questionnaires. We also examined longitudinal responses among HCW who completed the questionnaires multiple times.

All analyses were conducted with a two-sided statistical significance level of *P* < .05 using R statistical software (version 3.4.2).

## Results

### Participant characteristics

**Fig 1:**
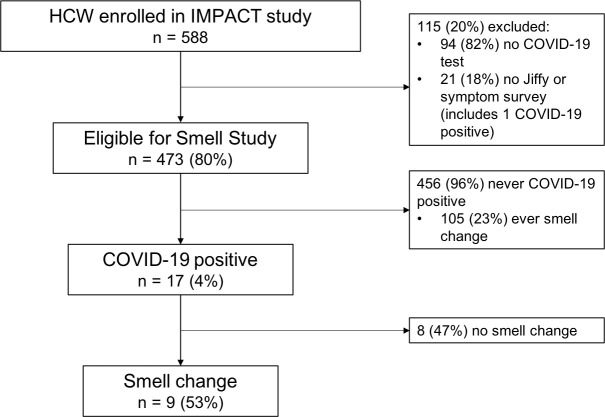
Study flow diagram. Flow diagram of participants and reported changes in smell by COVID-19 status. Abbreviations: HCW, healthcare workers; IMPACT, Implementing Medical and Public Health Action against Coronavirus (CT).

588 HCW were recruited and consented in the IMPACT study between March 31 and July 7, 2020, of whom 473 (80%) were eligible for the smell sub-study, while 115 (20%) were excluded because they did not undergo SARS-CoV-2 testing or they did not submit either a Yale Jiffy or symptom survey (**Fig 1**). Within the sub-study population, 373 (79%) participants were female; the mean (SD) age was 37.5 (11.2) years; 375 (79%) were white non-Hispanic/Latino; 261 (55%) were registered nurses (RNs) and 98 (21%) were medical doctors (MDs) (**Table 1**). Compared to HCW included in this analysis, IMPACT HCW ineligible for the sub-study were more likely to be Black, Asian, or other ethnicity (*P* < .001) and have higher BMI (*P* = 0.004) (**S3 Table**).

**Table 1:** Baseline characteristics of participants in the smell sub-study.

	SARS-CoV-2 positive HCW (*n* = 17)	SARS-CoV-2 negative HCW (*n* = 456)	*P* value^a^	aOR (95% CI)^b^
Demographics				
Age, y	30.0 (26.0, 48.0)	34.5 (29.0, 44.0)	0.47	--
Female sex	15 (88)	358 (79)	0.54	--
Ethnicity			0.84	--
White	16 (94)	359 (79)		
Black	0 (0)	15 (3)		
Hispanic	0 (0)	37 (8)		
Asian	1 (6)	36 (8)		
Other	0 (0)	9 (2)		
BMI, kg/m^2^	25.0 (22.2, 35.2)	24.7 (22.7, 29.1)	0.74	--
Occupation			0.01	--
RN	15 (88)	246 (54)		
MD	0 (0)	98 (21)		
Other	2 (12)	112 (25)		
Number of surveys completed				
Symptom survey	10.0 (6.0, 22.0)	22.0 (10.0, 34.0)	0.08	--
Yale Jiffy^c^	1.5 (1.0, 4.5)	8 (2.0, 24.0)	0.03	--
Smell loss				
Yale Jiffy	5/9 (56)	43/304 (14)	0.005	--
Symptom survey	8/17 (47)	83/456 (18)	0.008	--
Either survey	9/17 (53)	105/456 (23)	0.009	4.52 (1.61, 13.3)

Data are presented as median (IQR) for continuous variables and no. (%) for categorical variables.

Abbreviations: BMI, body mass index; CI: confidence interval; HCW, healthcare workers; MD, medical doctor; aOR, adjusted odds ratio; RN, registered nurse.

^a^ Unadjusted *P* values are Wilcoxon rank sum test (continuous variables) or Fisher’s exact test (categorical variables).

^b^ Adjusted odds ratios and 95% confidence intervals for symptoms were obtained from separate logistic regression models, each adjusted for age, sex, BMI, and number of symptom surveys completed.

^c^ *n* = 9 for SARS-CoV-2 positive HCW; *n* = 304 for SARS-CoV-2 negative HCW.

HCW included in the sub-study completed the daily symptom questionnaire, which included parosmia and hypogeusia screening, a median 23 times per HCW (IQR: 10, 34). Among the 313 (66%) HCW who completed the Yale Jiffy at least once, HCW completed a median of 10 (IQR: 3, 28) Jiffy questionnaires.

5771 SARS-CoV-2 RT-qPCR tests were performed on 473 HCW in the sub-study (median = 11 tests per HCW) between March 31 and July 7. Of these, 17 (3.6%) HCW tested positive for SARS-CoV-2.

### HCW who tested positive for SARS-CoV-2 were more likely to report smell loss

**Fig 2:**
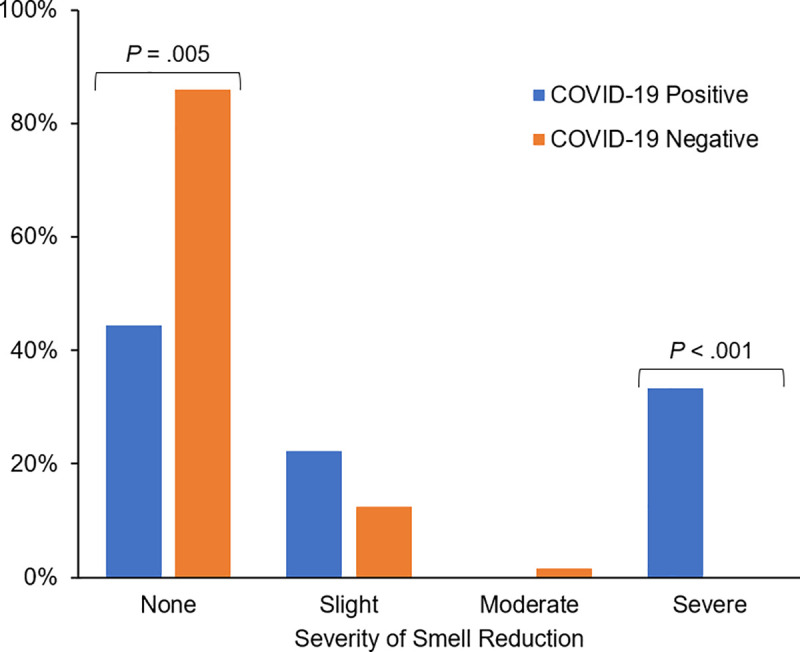
Self-reported smell loss by COVID status. Comparison of the proportions of self-reported severity of smell loss on the Yale Jiffy by COVID-positive and COVID-negative healthcare workers. *P* value is a Fisher’s exact test of independence.

The demographic characteristics of SARS-CoV-2-positive HCW were similar to those of SARS-CoV-2-negative HCW (**Table 1**). Nine of the 17 (53%) SARS-CoV-2-positive HCW completed the Yale Jiffy at least once. SARS-CoV-2-positive HCW were more likely to report categorical smell loss on the Jiffy, with 5/9 (56%) SARS-CoV-2-positive HCW versus 43/304 (14%) SARS-CoV-2-negative HCW reporting smell loss (OR = 7.6, 95% CI: 2.0–29.4) (**Table 1**). The five SARS-CoV-2-positive HCW who reported categorical smell loss via the Jiffy had a mean (SD) reduction in smell of 5.8 (4.0)cm on a 10cm (0.0 to 10.0) scale; those indicating severe loss had a mean 8.3 (3.0)cm decrease compared to 2.2 (0.5)cm in those with slight smell loss. For individuals with any smell loss reported via Jiffy, SARS-CoV-2-positive HCW reported more severe smell loss than SARS-CoV-2-negative HCW (*P* < .001) (**Fig 2**). SARS-CoV-2-positive HCW reported severe (3/5, 60%) or slight 2/5, 40%) smell loss on a categorical scale, while SARS-CoV-2- negative HCW reported slight (38/43, 88%) or moderate (5/43, 12%) smell loss.

All 17 SARS-CoV-2-positive HCW eligible for the smell sub-study completed at least one daily symptom questionnaire. Eight (47%) reported parosmia versus 83/456 (18%) SARS-CoV-2-negative HCW (OR = 4.0, 95% CI: 1.5–10.7). Hypogeusia was reported in seven (41%) SARS-CoV-2-positive HCW and 104/456 (23%) SARS-CoV-2-negative HCW (OR = 2.4, 95% CI: 0.7, 7.1) (**Table 1**). Six of those seven also reported smell loss.

**Fig 3:**
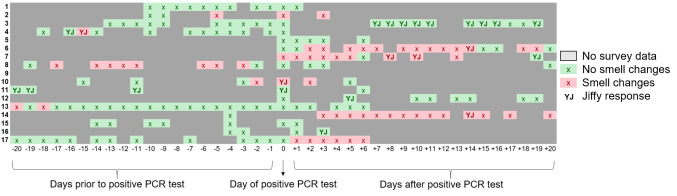
Chronology of smell changes among COVID-positive healthcare workers relative to the day of positive test. Red boxes indicate smell change was reported via either the symptom survey or Yale Jiffy. Green boxes indicate no smell change reported. Solid gray boxes indicate there was no Yale Jiffy or symptom survey submitted for that day. Boxes with a “YJ” specifically indicate a Yale Jiffy response.

Relative to Day 0 (defined as the day of test positivity), the median timing of reported smell loss was Day -2 (Range, Day -20 to Day +3) among SARS-CoV-2-positive HCW reporting smell loss, with 6/9 (67%) reporting smell loss before test positivity (**Fig 3**). Of those who reported smell loss, 3/9 (33%) reported changes in smell as their first symptom; all 9 reported symptoms of COVID-19 in addition to changes in smell.

### Associations between smell symptoms and SARS-CoV-2 infection

Overall, in either the daily symptom questionnaire or Yale Jiffy, nine (53%) SARS-CoV-2-positive HCW reported smell loss or parosmia, compared to 105/456 (23%) SARS-CoV-2-negative HCW (OR = 3.7, 95% CI: 1.2, 11.5). After adjusting for age, sex, BMI, and number of symptom questionnaires, smell loss remained a significant predictor of SARS-CoV-2 infection (aOR = 4.5, 95% CI: 1.6, 13.3). Among other symptoms in the questionnaire, only dyspnea (aOR 7.3, 95% CI: 2.2, 22.4) and headache (aOR 6.1, 95% CI: 1.8, 28.4) were more strongly predictive of SARS-CoV-2 infection. In the matched analysis, unadjusted conditional logistic regression estimated a significant association between smell loss and SARS-CoV-2 infection (OR = 6.0, 95% CI 1.6–22.2) **(S4 Table and S1 Fig**).

### Associations between smell loss and neurological symptoms in SARS-CoV-2-positive HCW

**Table 2** summarizes findings among SARS-CoV-2-positive HCW stratified by smell loss as reported by either measure. Neurological symptoms as assessed by the daily symptom questionnaire, including headache, fatigue, and dizziness, were reported in all nine SARS-CoV-2-positive HCW reporting smell loss, and three (38%) who did not report smell loss. Prolonged neurological symptoms (> seven days after positive test) were reported in four (44%) SARS-CoV-2-positive HCW with smell loss versus one (13%) without smell loss. Three SARS-CoV-2-positive HCW, all of whom experienced smell loss, had significantly prolonged neurological symptoms (> 20 days after positive test).

**Table 2:** Characteristics of SARS-CoV-2 positive healthcare workers by reported smell change.

	Smell change (*n* = 9)	No smell change (*n* = 8)	*P* value^a^
Cycle threshold, mean (SD)	27.7 (4.9)	30.2 (3.5)	0.28
Age	34 (30, 59)	26.5 (25.8, 33.8)	0.054
Female sex	9 (100)	6 (75.0)	0.21
Non-white race	0 (0)	1 (12.5)	0.47
BMI	26.6 (22.3, 35.5)	23.7 (22.6, 27.7)	0.71
Smell change as presenting symptom	3 (33.3)	--	--
Any neurological symptoms^b^	9 (100)	3 (37.5)	0.009
Neurological symptoms > 7 days after positive test^b^	4 (44.4)	1 (12.5)	0.29

Data are presented as median (IQR) for continuous variables and no. (%) for categorical variables unless otherwise indicated.

Abbreviations: BMI, body mass index.

^a^ Unadjusted *P* values obtained from student’s *t* test (normally distributed continuous variables), Wilcoxon rank sum test (non-normally distributed continuous variables), and Fisher’s exact test (categorical variables).

^b^ Neurological symptoms include headache, dizziness, and fatigue.

## Discussion

In this prospective study of a high-risk HCW population, we assessed smell loss alongside routine viral testing, finding that HCW who acquired SARS-CoV-2 infection over the course of the study had significantly increased odds of reporting smell loss compared to SARS-CoV-2-negative HCW. This finding was consistent across bivariate (OR = 3.7), regression-adjusted (OR = 4.5), and matched (OR = 6.0) analyses. Likewise, SARS-CoV-2-positive HCW reported significantly more severe smell loss compared to SARS-CoV-2-negative HCW. Overall, our findings add to the accumulating evidence demonstrating the efficacy and feasibility of using prospective, self-administered, at-home assessments of smell sensitivity to track changes over time in a group of individuals at high risk for SARS-CoV-2 infection.

Our findings are consistent with recent research demonstrating robust associations between smell perturbations and SARS-CoV-2 infection. Specifically, smell and taste impairments are reported prior to (20%) or during (13%) hospitalization [22], typically lasting 4–17 days [23], and provide better predictive ability than fever or cough [24], two of the more commonly used symptoms to screen for COVID-19. Indeed, a large cross-sectional study found that sudden self-reported smell loss was the best predictive indicator of SARS-CoV-2 infection [25]. However, objective smell measures have been shown to be more sensitive than subjective self-report measures, which tend to underestimate prevalence of olfactory dysfunction in COVID-19 [8, 9, 13]. Our study extends these findings by uniquely assessing for prospective smell loss alongside viral screening in a high-risk group, all of whom were SARS-CoV-2-negative at study entry. Moreover, distortion in smell was a better predictor of SARS-CoV-2 infection than many other physical symptoms. Cough, headache, and dyspnea were also significantly associated with increased odds of SARS-CoV-2 infection in this study. However, these symptoms are commonly experienced in other respiratory infections and not specific symptoms for COVID-19, and therefore less useful than loss of smell for monitoring for COVID-19 among asymptomatic individuals.

Interestingly, we found an association between reduction in smell ability and neurological symptoms in HCW with SARS-CoV-2 infection. While the exact pathophysiology underlying COVID-19 related smell loss remains incompletely understood, the association between smell loss and other neurological symptoms suggests a potential common mechanism underlying these symptoms. A growing body of evidence has demonstrated the potential for SARS-CoV-2 to infect neurons [26, 27], and our results lend support to the hypothesis that smell loss may be related to central nervous system infection by SARS-CoV-2, consistent with studies reporting MRI abnormalities in the olfactory bulb in a subset of patients with COVID-19 related anosmia [28, 29].

### Limitations and future directions

Our study has several limitations. Despite performing almost 6,000 SARS-CoV-2 tests on nearly 500 HCW during the first peak of the COVID-19 pandemic in Connecticut, only 17 (3.6%) HCW tested positive, limiting the statistical power of our analysis. Nevertheless, we found large, significant effects. Smell sensitivity and loss using the Yale Jiffy were evaluated using both categorical responses and a VAS [30]. Indeed, those indicating severe loss reported an average of an 8.3cm decrease on the 10cm scale, compared to a 2.2cm reduction in those indicating slight loss, suggesting categorical responses are sufficient for identifying asymptomatic carriers. However, because many participants already had smell loss the first time they completed the VAS, we were not able to detect any changes. Consistent daily ratings of a household item could prove useful for further evaluating the magnitude of loss but would require further instructions and possibly practice using the scale. Adopting the generalized labeled magnitude scale (gLMS), a specialized line scale with semantic labels at empirically derived intervals, may also improve outcomes, as this scale is less subject to floor and ceiling effects [31]. However, it requires some training and practice. Finally, we note that there are limitations inherent with the use of household items, such as unknown stability across time and the use of variable stimuli across participants. However, since commercial products are made for stability and the primary comparisons of interest are within-subject, the use of household items provides an opportunity for participants to evaluate an actual odor, rather than depending only on memory.

## Conclusions

In our study, self-reported changes in smell perception were predictive of SARS-CoV-2 infection in a healthcare worker population. At-home smell assessments should be considered for non-invasive screening of groups that are at high risk for COVID-19.

## Supporting information

S1 Fig Results of matching on age, sex, ethnicity, BMI, and number of questionnaires. Results of matching on age, sex, profession, ethnicity, and number of symptom questionnaires completed for HCW reporting smell on either survey (A, B), the daily symptom survey only (C, D), and the Yale Jiffy only (E, F). A, C, and E show the change an absolute standardized difference in means between the unmatched (“All Data”) and matched datasets. B, D, and F compare the distributions, as histograms, of the propensity scores between HCW with smell loss (“Treated”) and those without (“Control”) both before (“Raw”) and after (“Matched”) matching. In all cases, matching resulted in a decrease in the absolute standardized difference for the overall distance measure and a more similar distribution (as shown by histograms) of propensity scores.
(TIF)

S1 Table The Yale Jiffy survey. (DOCX)

S2 Table Parosmia questionnaire. Adapted from Landis BN, Frasnelli J, Croy I, Hummel T. Evaluating the clinical usefulness of structured questions in parosmia assessment. *Laryngoscope*. 2010;120(8):1708.
(DOCX)

S3 Table Characteristics of IMPACT study HCW who were included and excluded from the smell sub-study. Data are presented as median (IQR) for continuous variables and no. (%) for categorical variables. Abbreviations: BMI, body mass index; HCW, healthcare workers; IMPACT, Implementing Medical and Public Health Action against Coronavirus (CT); MD, medical doctor; RN, registered nurse. ^a^ Unadjusted *P* values are Wilcoxon rank sum test (continuous variables) or Fisher’s exact test (categorical variables).
(DOCX)

S4 Table Association between smell loss and SARS-CoV-2 infection after matching on age, sex, ethnicity, BMI, and number of questionnaires. Abbreviations: CI, confidence interval. OR, odds ratio. ^a^ Odds ratios are presented for the association between smell loss and SARS-CoV-2 infection as calculated from unadjusted conditional logistic regression after matching on demographics and symptom questionnaire participation.
(DOCX)
