# Supplementary figures and images for: Tracking smell loss to identify healthcare workers with SARS-CoV-2 infection
Source: PLoS One. 2021 Mar 3;16(3):e0248025. doi: 10.1371/journal.pone.0248025 (PMC7928484; doi:10.1371/journal.pone.0248025)

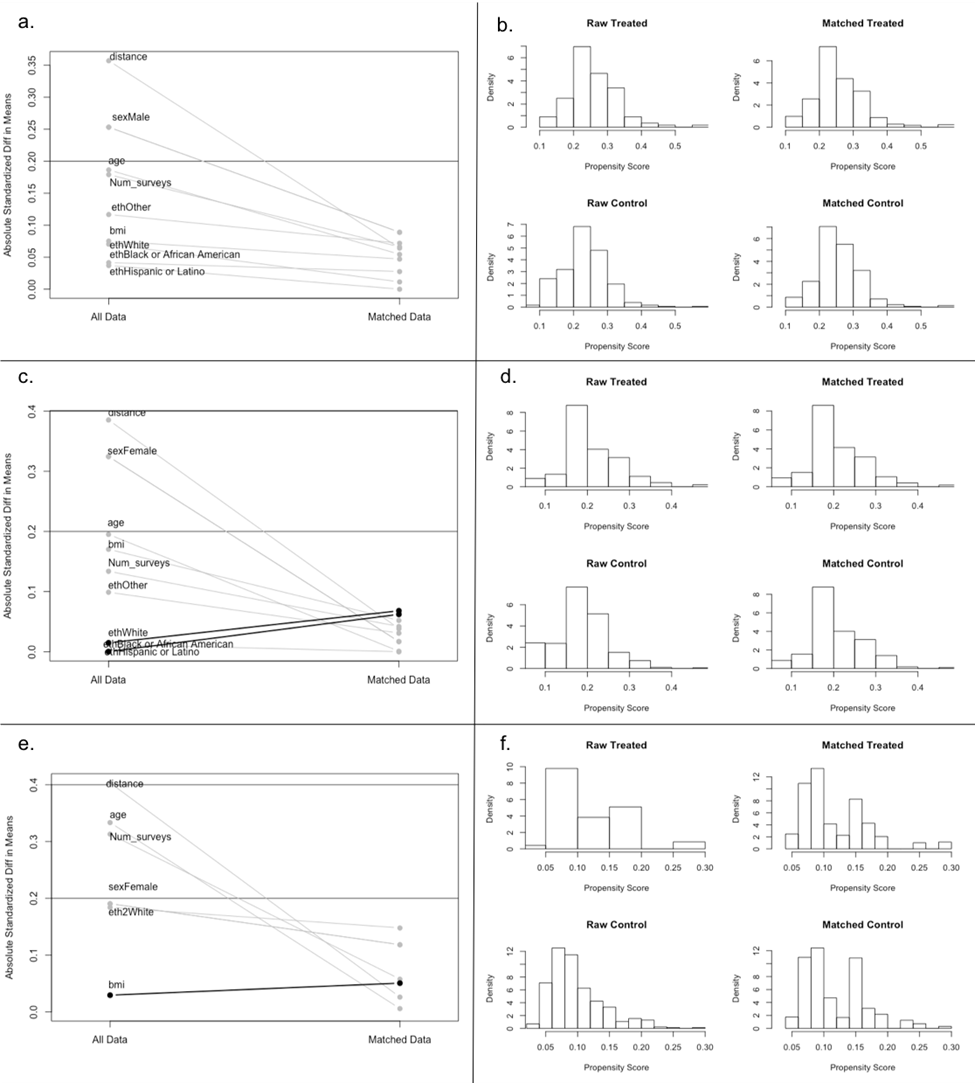

Supplement: S1 Fig — Results of matching on age, sex, profession, ethnicity, and number of symptom questionnaires completed for HCW reporting smell on either survey (A, B), the daily symptom survey only (C, D), and the Yale Jiffy only (E, F). A, C, and E show the change an absolute standardized difference in means between the unmatched (“All Data”) and matched datasets. B, D, and F compare the distributions, as histograms, of the propensity scores between HCW with smell loss (“Treated”) and those without (“Control”) both before (“Raw”) and after (“Matched”) matching. In all cases, matching resulted in a decrease in the absolute standardized difference for the overall distance measure and a more similar distribution (as shown by histograms) of propensity scores. (TIF) [file pone.0248025.s001.tif]
