# Supplementary material for: Tracking smell loss to identify healthcare workers with SARS-CoV-2 infection
Source: PLoS One. 2021 Mar 3;16(3):e0248025. doi: 10.1371/journal.pone.0248025 (PMC7928484; doi:10.1371/journal.pone.0248025)
Supplement: S1 Table — (DOCX) [file pone.0248025.s002.docx]

**S1 Table. The Yale Jiffy survey**

| **Initial questions** | **Answer options** |  |
| --- | --- | --- |
| How would you rate your ability to smell | Poor, average, good, very good |  |
| Have you noticed a reduction in your sense of smell in the past week? | None, slight, moderate, severe |  |
| Please indicate how much reduction (if any) you believe you have experienced in the past week | 100mm scale (“no reduction at all” to “extreme reduction”) |  |
| **Smell test instructions** | **Smell test questions** | **Smell test answer options** |
| Please find a jar of peanut butter (or other nut butter). If you have any nut allergies, please use jam or jelly. | What do you have? | Peanut butter, other nut butter, jelly, jam, other |
| Open it and bring it to about 1 inch from your nose. Please sniff. | Do you smell it? | Yes/No |
|  | How strong does it smell? | 100mm scale (“no sensation” to “strongest sensation imaginable”) |
|  | Does it smell different from normal? | Yes/No |
|  | Please indicate how different it is | 100mm scale (“no different” to “completely different”) |
| Please find a jar of vinegar (white vinegar is best) or a muscle balm (e.g., Tiger balm or Bengay). | What do you have? | Vinegar, Tiger balm, Bengay, nail polish remover, rubbing alcohol, other |
| Open it and bring it to about 1 inch from your nose. Please sniff. | Do you feel a sensation of irritation (e.g., burning, stinging, harshness) in your nose or throat? | Yes/No |
|  | How strong was the sensation? | 100mm scale (“no irritation” to “strongest irritation imaginable”) |
|  | Does it smell different from normal? | Yes/No |
|  | Please indicate how different it is | 100mm scale (“no different” to “completely different”) |
