# Supplementary material for: Tracking smell loss to identify healthcare workers with SARS-CoV-2 infection
Source: PLoS One. 2021 Mar 3;16(3):e0248025. doi: 10.1371/journal.pone.0248025 (PMC7928484; doi:10.1371/journal.pone.0248025)
Supplement: S2 Table — Adapted from Landis BN, Frasnelli J, Croy I, Hummel T. Evaluating the clinical usefulness of structured questions in parosmia assessment. Laryngoscope. 2010;120(8):1708. (DOCX) [file pone.0248025.s003.docx]

**S2 Table.** **Parosmia questionnaire**

| **How often are you bothered by any of the below?** | **Always** | **Often** | **Rarely** | **Never** |
| --- | --- | --- | --- | --- |
| 1. Food tastes different than it should because of a problem with odors. | 1 | 2 | 3 | 4 |
| 2. I always have a bad smell in my nose, even if no odor source is present. | 1 | 2 | 3 | 4 |
| 3. Odors that are pleasant to others are unpleasant to me. | 1 | 2 | 3 | 4 |
| 4. The biggest problem is not that I do not or only weakly perceive odors, but that they smell different than they should. | 1 | 2 | 3 | 4 |

Adapted from Landis BN, Frasnelli J, Croy I, Hummel T. Evaluating the clinical usefulness of structured questions in parosmia assessment. *Laryngoscope*. 2010;120(8):1708.
