# Supplementary material for: Tracking smell loss to identify healthcare workers with SARS-CoV-2 infection
Source: PLoS One. 2021 Mar 3;16(3):e0248025. doi: 10.1371/journal.pone.0248025 (PMC7928484; doi:10.1371/journal.pone.0248025)
Supplement: S3 Table — Data are presented as median (IQR) for continuous variables and no. (%) for categorical variables. Abbreviations: BMI, body mass index; HCW, healthcare workers; IMPACT, Implementing Medical and Public Health Action against Coronavirus (CT); MD, medical doctor; RN, registered nurse. a Unadjusted P values are Wilcoxon rank sum test (continuous variables) or Fisher’s exact test (categorical variables). (DOCX) [file pone.0248025.s004.docx]

**S3 Table. Characteristics of IMPACT study HCW who were included and excluded from the smell sub-study**

|  | IMPACT HCW included in smell sub-study  (*n* = 473) | IMPACT HCW excluded from smell sub-study  (*n* = 115) | *P* value^a^ |
| --- | --- | --- | --- |
| Demographics |  |  |  |
| Age, y | 34.0 (29.0, 44.0) | 34.0 (28.0, 42.0) | 0.62 |
| Female sex | 374 (79) | 91 (79) | 1 |
| Ethnicity |  |  | <0.001 |
| White | 375 (79) | 67 (58) |  |
| Black | 15 (3) | 18 (16) |  |
| Hispanic | 37 (8) | 8 (7) |  |
| Asian | 37 (8) | 14 (12) |  |
| Other | 9 (2) | 8 (7) |  |
| BMI, kg/m^2^ | 24.7 (22.7, 29.1) | 27.1 (23.4, 31.6) | 0.004 |
| Occupation |  |  | 1 |
| RN | 261 (55) | 64 (56) |  |
| MD | 97 (21) | 23 (20) |  |
| Other | 115 (24) | 28 (24) |  |

Data are presented as median (IQR) for continuous variables and no. (%) for categorical variables.

Abbreviations: BMI, body mass index; HCW, healthcare workers; IMPACT, Implementing Medical and Public Health Action against Coronavirus (CT); MD, medical doctor; RN, registered nurse.

^a^ Unadjusted *P* values are Wilcoxon rank sum test (continuous variables) or Fisher’s exact test (categorical variables).
