# Supplementary material for: Tracking smell loss to identify healthcare workers with SARS-CoV-2 infection
Source: PLoS One. 2021 Mar 3;16(3):e0248025. doi: 10.1371/journal.pone.0248025 (PMC7928484; doi:10.1371/journal.pone.0248025)
Supplement: S4 Table — Abbreviations: CI, confidence interval. OR, odds ratio. a Odds ratios are presented for the association between smell loss and SARS-CoV-2 infection as calculated from unadjusted conditional logistic regression after matching on demographics and symptom questionnaire participation. (DOCX) [file pone.0248025.s005.docx]

**S4 Table. Association between smell loss and SARS-CoV-2 infection after matching on age, sex, ethnicity, BMI, and number of questionnaires**

|  | **Either Yale Jiffy or symptom survey** | **Symptom survey only** | **Yale Jiffy only** |
| --- | --- | --- | --- |
| *n* reporting smell loss | 114 | 89 | 47 |
| *n* matched controls who never reported smell loss | 228 | 178 | 94 |
| OR (95% CI)^a^ | 6.0 (1.6-22.2) | 3.2 (1.1-9.8) | 5.0 (0.97-25.8) |

Abbreviations: CI, confidence interval. OR, odds ratio.

^a^Odds ratios are presented for the association between smell loss and SARS-CoV-2 infection as calculated from unadjusted conditional logistic regression after matching on demographics and symptom questionnaire participation.
